# Removal of oil spills by novel developed amphiphilic chitosan-*g*-citronellal schiff base polymer

**DOI:** 10.1038/s41598-021-99241-9

**Published:** 2021-10-06

**Authors:** Ahmed Mohamed Omer, Basant Yossry Eweida, Tamer Mahmoud Tamer, Hesham M. A. Soliman, Safaa Mohamed Ali, Ahmed Amin Zaatot, Mohamed Samir Mohy-Eldin

**Affiliations:** 1 Polymer Materials Research Department, Advanced Technology and New Materials Research Institute (ATNMRI), City of Scientific Research and Technological Applications (SRTA-City), New Borg El-Arab City, P. O. Box: 21934, Alexandria, Egypt; 2 Modeling and Simulation Department, Advanced Technology and New Materials Research Institute, City of Scientific Research and Technological Applications (SRTA-City), New Borg El-Arab City, Alexandria, 21934 Egypt; 3 Nanotechnology and New Composite Materials Department Advanced Technology and New Materials Research Institute (ATNMRI), City of Scientific Research and Technological Applications (SRTA-City), New Borg El-Arab City, Alexandria, 21934 Egypt; 4 Nucleic Acid Research Department, Genetic Engineering and Biotechnology Research Institute (GEBRI), City for Scientific Research and Technological Applications (SRTA-City), New Borg El-Arab, Alexandria, 21934 Egypt; 5 Chemical Engineering Department, Faculty of Engineering, Alexandria University, Alexandria, Egypt

**Keywords:** Environmental sciences, Chemistry, Materials science

## Abstract

A novel chitosan grafted citronellal (Ch-Cit) schiff base amphiphilic polymer was developed for the adsorptive removal of oil spills. The chemical structure was verified by FT-IR spectroscopy and ^1^H NMR spectrometer, while the morphological changes and surface area were investigated by SEM and BET analysis tools. The amphiphilic character of Ch-Cit schiff base was controlled through variation of the grafting percentage (G%) of citronellal from 11 to 61%. Dramatic changes in the ion exchange capacity (IEC), solubility and water uptake profiles were established, while the oil adsorption capacity was founded in direct relation with the G (%) of citronellal. Operational conditions such as oil amount, adsorption time, adsorbent dose and agitation speed were investigated. The developed Ch-Cit schiff base exhibited a higher surface area (115.94 m^2^/g) compared to neat chitosan (57.78 m^2^/g). The oil adsorption capacity of the Ch-Cit schiff base was greatly improved by 166% and 120% for light crude and heavy crude oil, respectively. Finally, the adsorption process was optimized using response surface methodology (RSM).The results substantiate that the amphiphilic Ch-Cit schiff base could be efficiently applied as a low-cost oil-adsorbent for the removal of crude oil spills from sea-water surfaces.

## Introduction

Currently, several technologies have been developed for water remediation; for instance, natural materials are widely recognized by the scientific community as high potential substrates for many purposes^1,2^. In particular, functionalization processes have improved the affinity of the developed materials towards several classes of pollutants such as heavy metals, organic compounds, endocrine disrupting compounds, dyes and oil spills^3–6^. It is well known that the crude oils have gained its importance for various industrial sectors^7^. However, the processes and activities related to the crude oil and its derivatives may result in oil spillage^8^, which possesses crucial environmental threats to the aquatic system and human life^9^. In addition, overcoming these difficulties take a long time and much effort^10^. Natural sorbent materials have attracted much interest during the last decade due to their unique features including biodegradable, reliable and available in nature and low-cost production^11,12^. The oil sorption process goes through multi-sorption steps (i.e. oil diffusion, oil entrapment and finally oil droplets agglomeration) depending on the interactions incident on the sorbent surface, internal structure of sorbent and pores of the sorbent^13,14^. Among these natural sorbents, chitosan (Ch) is a naturally occurring biopolymer which composed of ß-(1 → 4)-2-acetamido-2-deoxy-d-glucopyranose and ß-(1 → 4)-2-amino-2-deoxy-d-glucopyranose units^15^. Chitosan can be obtained via deacetylation of chitin biopolymer which extracted from marine sources such as crabs and shrimp shells^16^. Owing to its outstanding characteristics such as eco-friendly, bio-degradability and ease of functionalization chitosan has been effectively applied in biomedical, industrial and water treatment fields^17–19^. Therefore, chitosan has been evaluated its capability for the adsorptive removal of various pollutants such as heavy metals, organic dyes, pharmaceutical residues and oil spills^20,21^. Nevertheless, the higher hydrophilicity and the limited surface area are considered the major drawbacks of the oil adsorbents-based chitosan^22^. Thus, several modification processes such as composite formation^18^, grafting^23^ and crosslinking^24^ have been conducted for native chitosan biopolymer to improve its adsorption tendency towards various oils types.

This study focused on the development of novel amphiphilic chitosan-*g*-citronellal (Ch-Cit) schiff base as a low-cost oil adsorptive material. Herein, the new schiff base derivative was synthetized via a click grafting technique through coupling of the aldehyde groups of citronellal with the present amine groups of chitosan. The developed Ch-Cit schiff base is expected to acquire higher hydrophobicity relative to the native chitosan due to the hydrophobic character of citronellal as well as consumption of the hydrophilic chitosan’ amine group during the formation of schiff base. Furthermore, additional amine groups are expected to engage in the formation of three dimensions structure via chemical crosslinking with glutaraldehyde. The crosslinking step could also reduce the hydrophilicity of the chitosan backbone in addition to induce pores structure. Accordingly, the developed amphiphilic Ch-Cit schiff base has the possibility to adsorb oils through its hydrophobic citronellal grafted moiety as well as through its pores structure. The novel schiff base derivative characterized using various characterization tools. The amphiphilic Ch-Cit schiff base derivative was evaluated its affinity towards the adsorptive removal of different oil types under different operational adsorption conditions. Besides, the response surface method (RSM) technique was applied to recognize the optimum operating conditions for the adsorptive removal of oil spills.

## Experimental section

### Materials

Sodium hydroxide pellets (Assay 99%, M_wt_ = 40 g/mol) was obtained from by EL. pharanae co. (Egypt). Sodium chloride (Assay 99%, M_wt_ = 58.44 g/mol), Sulfuric acid (Purity 95–97%) were purchased from El-Gomhouria Co. (Egypt). Acetic acid (Purity 99.8%, M_wt_ = 60.05 g/mol), Glutaraldehyde (GA; 25%; 99%), Citronellal (Purity ≥ 95%, M_wt_ = 154.25 g/mol), Mineral oil (Density 0.838 g/ml at 25 °C) were acquired from Sigma-Aldrich chemical LTD (Germany). Ethanol (Purity 99%), Hydrochloric acid (Assay 37%) were brought from El-Nasr Co. (Egypt). Shrimp skeletons were provided from commercial resource in Alexandria (Egypt). Diesel oil and Kerosene oil were supplied from local gas station, (Egypt).Crude oil samples: Two crude oils, namely; light crude (LC) and heavy crude (HC) oils were supplied from Belayem Petroleum Co. (Egypt).

## Methods

### Preparation of chitosan (Ch)

Chitin was firstly extracted via de-mineralization of the crushed shrimp shells according to the published procedure^25^. Next, the resultant chitin was de-acetylated under alkaline conditions at 100–120 °C for 12 h for removing the acetyl groups^26^. The produced chitosan was further purified by dissolving it in 2% (w/v) of acetic acid solution followed by filtration, washing and finally dried at 60 °C. The degree of de-acetylation was estimated according to the author’s previous studies via FT-IR and potentiometric titration methods, and recorded 94.4% and 93.15%, respectively^27^.

### Preparation of chitosan-*g*-citronellal schiff base (Ch-Cit)

**Figure 1:**
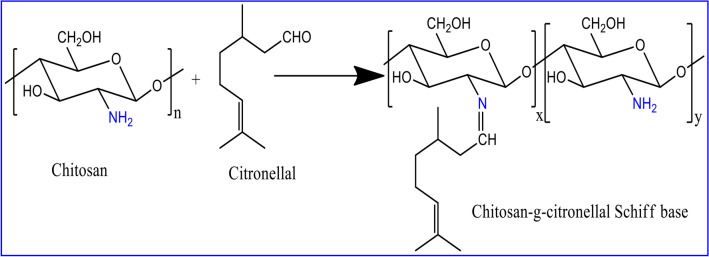
Proposed mechanism for the synthesis of chitosan-*g*-citronellal schiff base (Ch-Cit).

Previously purified chitosan (Ch; 0.4 g) was dissolved in 20 ml of 2% acetic acid and stirred at room temperature for 6 h. The resulting viscous solution was filtered through a cheese cloth to remove un-dissolved particles. An accurate 10 ml of ethanol was added to chitosan solution under stirring at room temperature. Next, 5 ml of ethanol containing a definite amount of citronellal (Cit) was added the solution under stirring to have a homogenous solution. This mixture was stirred for 6 h at 80 °C and followed by addition of 100 µl of GA with continuous stirring for 1 h. The formed product was immersed into excess of 1 M of sodium hydroxide solution. The precipitate was filtered and washed with distilled water and ethanol several times to remove un-reacted citronellal, and followed by drying overnight in a vacuum oven at 60 °C. One mole of chitosan reacted with 0.43, 0.85, 1.7, 2.55, 3.4 and 4.25 mol of citronellal, which coded according to their grafting percentage values as Ch-Cit (11.25% G), Ch-Cit (33.25% G), Ch-Cit (46.45% G), Ch-Cit (50.0% G), Ch-Cit (56.25% G) and Ch-Cit (61.0% G), respectively. A schematic representation for the preparation chitosan-g-citronellal schiff base (Ch-Cit) was depicted in Fig. 1. The grafting percentage (G%) of citronellal onto chitosan backbone was calculated according to the following equation^28^.

1 $${\text{G}}(\% ) = \frac{{{{{\text{W}}_{1}}  - {{\text{W}}_{0}}} }}{{{\text{W}}_{0} }} \times 100, $$

where, W_0_ and W_1_ represent weights of chitosan and chitosan-*g*-citronellal schiff base, respectively.

### Materials characterization

The chemical structures of chitosan and its citronellal grafted schiff base were verified by Fourier Transform Infrared Spectroscopy (FT-IR; Shimadzu—8400 S, Japan) and Nuclear Magnetic Resonance Spectrometer (^1^HNMR; JEOL 500 MH, Japan). The thermal properties were inspected under nitrogen atmosphere by Thermal Gravimetric Analyzer (TGA; Shimadzu-50, Japan), while the temperature was raised up to 800 ^○^C. The surface morphology of the developed materials was examined using a Scanning Electron Microscope (SEM; Joel JSM 6360LA, Japan). Before SEM observation, all samples were fixed on aluminum stubs and vacuum coated with gold. Besides, the specific surface area of the developed materials was measured by Bruner–Emmett–Teller method (BET—Beckman coulter SA3100).

#### Determination of ion exchange capacity

The ion exchange capacity (IEC) of both chitosan (Ch) and amphiphilic chitosan-*g*-citronellal schiff base (Ch-Cit) derivatives were determined according to the reported method with a slight modification^13^. In brief, the tested sample was soaked at room temperature in 0.1 M H_2_SO_4_ solution for 2 h. The mixture was filtered and titrated against NaOH solution, while the ion exchange capacity (IEC) was calculated according to the following equation:

2 $$ {\text{IEC}}\,({\text{m}}_{{{\text{eq}}}} /{\text{g}}) = \frac{{\left( {{\text{V}}_{2}  - {\text{V}}_{1} } \right){\text{N}}}}{{\text{W}}}, $$

where V_2_ and V_1_ are the volumes of NaOH required for completion the neutralization of H_2_SO_4_ in the absence and presence of tested sample, respectively, while N and W are the normality of NaOH and weight of tested sample.

#### Solubility and water uptake studies

Solubility of the developed materials was investigated via immersing an accurate quantity of tested sample in an aqueous solution with the pH range of 3–8 form at room temperature. The solubility (%) was estimated weighing the residual amount of un-dissolved sample matrix. Water uptake estimation was performed also by placing a 0.1 g of tested sample in 10 ml of distilled water. After fixed time, the sample was then filtered off, carefully bolted with a filter paper and weighed. The water uptake (WU) was calculated by applying the following equation:

3 $$   {\text{WU}}\,({\text{g}}/{\text{g}}) = \frac{{\left( {{\text{M}}_{{\text{t}}}  - {\text{M}}_{0} } \right)}}{{{\text{M}}_{0} }}, $$

where, M_t_ is the weight of the swollen sample at time t and M_0_ is the initial weight of the dried sample**.**

#### Oil uptake capacity measurements

A known weight of the examined sample (0.1 g) was placed in a closed glass tube containing 10 ml of oil for a definite time. Next, the swollen sample was separated carefully and weighted in a closed electronic balance. Varies oils types were used namely; mineral oil, kerosene, diesel oil, light crude oil (LC oil) and heavy crude oil (HC oil). The oil uptake capacity was calculated by applying the following equation:

4 $${\text{Oil uptake capacity}}\,({\text{g}}/{\text{g}}) = \frac{{\left( {{{\text{W}}_{t}}  - {{\text{W}}_{0}} } \right)}}{{{\text{W}}_{0} }}, $$

where W_t_ and W_0_ represent the weight of the oil-sorbed sample and the weight of dried sample, respectively**.**

#### Batch oil adsorption studies

The adsorption of oil was performed based on the Standard Test Method for adsorption performance of adsorbent materials (ASTM F726-99) using oil-artificial sea water system (salinity 3.5% NaCl)^29^. The oil adsorption process was conductedusing two crude oils namely; light crude oil (LCO) and heavy crude oil (HCO). Several adsorption conditions including adsorbent dose (0.1–0.5 g), oil dosage (0.25–2.5 g) and agitation speed (50–250 rpm) were studied.

All experiments were conducted in triplicate (n = 3), and the obtained data were presented as the means corrected by standard deviation (± S.D.).

## Results and discussion

### Characterization of chitosan-*g*-citronellal schiff base

#### FT-IR

**Figure 2:**
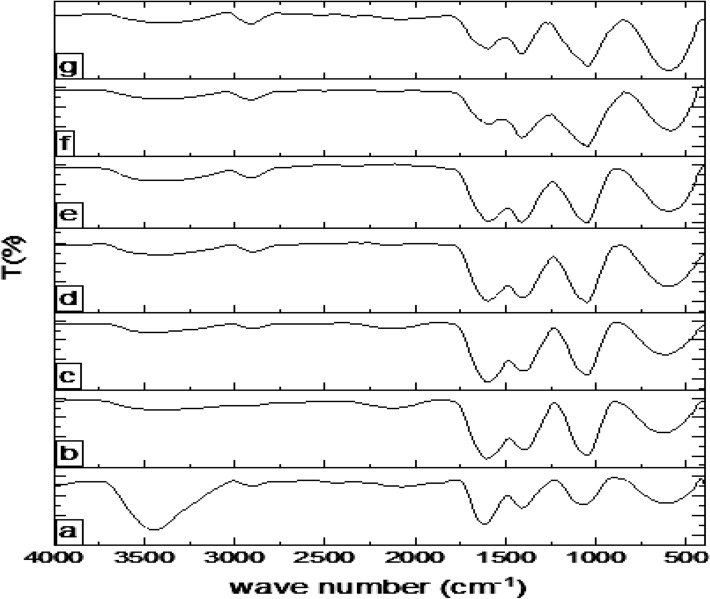
FT-IR spectra of (a) Ch, (b) Ch-Cit (11.25% G), (c) Ch-Cit (33.25% G), (d) Ch-Cit (46.45% G), (e) Ch-Cit (50.0% G), (f) Ch-Cit (56.25% G) and (g) Ch-Cit (61.0% G) schiff bases.

Figure 2 represents FT-IR spectra of the neat chitosan (Ch) and chitosan-*g*-citronellal schiff base (Ch-Cit) derivatives with different grafting percentages. The IR spectrum of native chitosan (Fig. 2a) demonstrates the typical bands of chitosan function groups^30^. A broad band between 3425 cm^−1^ was observed which matching to the stretching vibration of NH_2_ and OH groups that distributed along the polymer backbone. The weak absorption peak at 2895 cm^−1^ is associated with the C–H stretching for methyl and methylene groups, while the characteristic peak at 1624 cm^−1^ could be assigned to the C=O stretching. In addition, the observed band at 1070 cm^−1^ could be ascribed to the stretching of the C–O–C bridge^31^. Structural analysis of citronellal by FT-IR spectrophotometer showed the identical spectrum with ν_max_: 1724 (s, C=O aldehyde), 2870 and 2716 (w, C–H aldehyde), 2924 (s, C–H sp^3^), 1643 (w, C=C), 1450 (m, –CH_2_–) and 1381 cm^−1^ (m, –CH_3_)^32^. Chitosan-*g*-citronellal schiff base shows significant changes compared with the native chitosan (Fig. 2b–g). The major differences are: (a) a strong absorption peaks at around 1606 cm^−1^ corresponding to the C=N stretching which formed between the aldehyde groups of citronellal and chitosan’ amine groups. (b) absorption band at around 2920 cm^−1^ for Ch-Cit schiff base of –CH stretching and (c) absorption band at around 1415 cm^−1^ for the merger between –NHCO (amide III) band at 1395 cm^−1^ of chitosan and –CH_2_ band at 1450 cm^−1^ and –CH_3_ band at 1381 cm^−1^ of the grafted citronellal^32^.

#### NMR analysis

**Figure 3:**
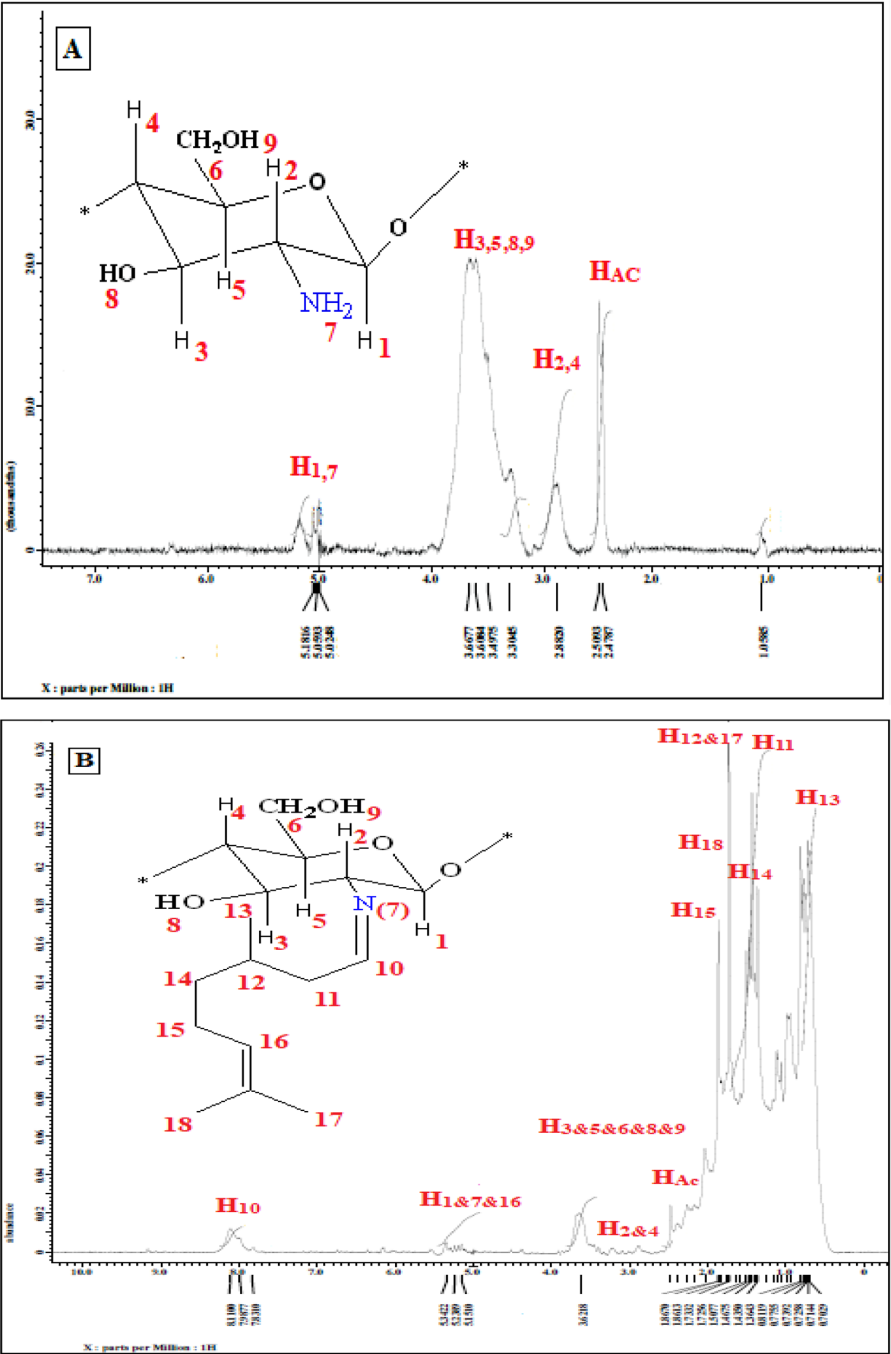
H^1^ NMR of (**A**) chitosan and (**B**) chitosan-*g*-citronellal schiff base.

^1^H NMR spectrum was employed to investigate the chemical structure of the new synthesized chitosan-*g*-citronellal schiff base as well as to estimate the degrees of deacetylation and substitution. All NMR spectra were accumulated under identical conditions using power gated Waltz decoupling with 25-degree measurement pulse and 1 s pre-pulse delay. Figure 3A shows the characteristic chemical shift of proton in the amino glucose ring of chitosan as δ 2.5 ppm from the three methyl H atoms (GlcNAc), δ 2.88 ppm from H2 (GlcN). Several overlapping signals from δ 3.4 to 3.60 ppm are detected which refer to H3–H6 that connected to the non-anomeric C3–C6 carbons in the glucopyranose ring, and ca. δ 5.1 ppm from anomeric proton (H1)^33–35^. Moreover, the deacetylation degree of chitosan was calculated and recorded a maximum value of 95.4%^36^. Besides, chitosan-*g*-citronellal (Ch-Cit) schiff base (Fig. 3B) demonstrated a complicated chart as its new signals overlapped with the neat chitosan polymer. The characteristic peak at δ 7.9 to 8 ppm refers to the immune proton (H10). The new signal at 0.9–1 ppm could be attributed to the protons of methyl groups (H13). Furthermore, the observed new overlapped signals at δ 1.18–1.43 ppm refer to H11 and H14, while the multi signals at δ 1.7–1.8 could be related to methylene protons H12, H15.H17 and H18. The proton signal for unsaturated carbon (H16) was overlapped with H1 and H7 in chitosan at δ 5.1–5.2 ppm. The degree of substitution was determined and recorded 17.7% of citronellal substitution.

#### SEM analysis

**Figure 4:**
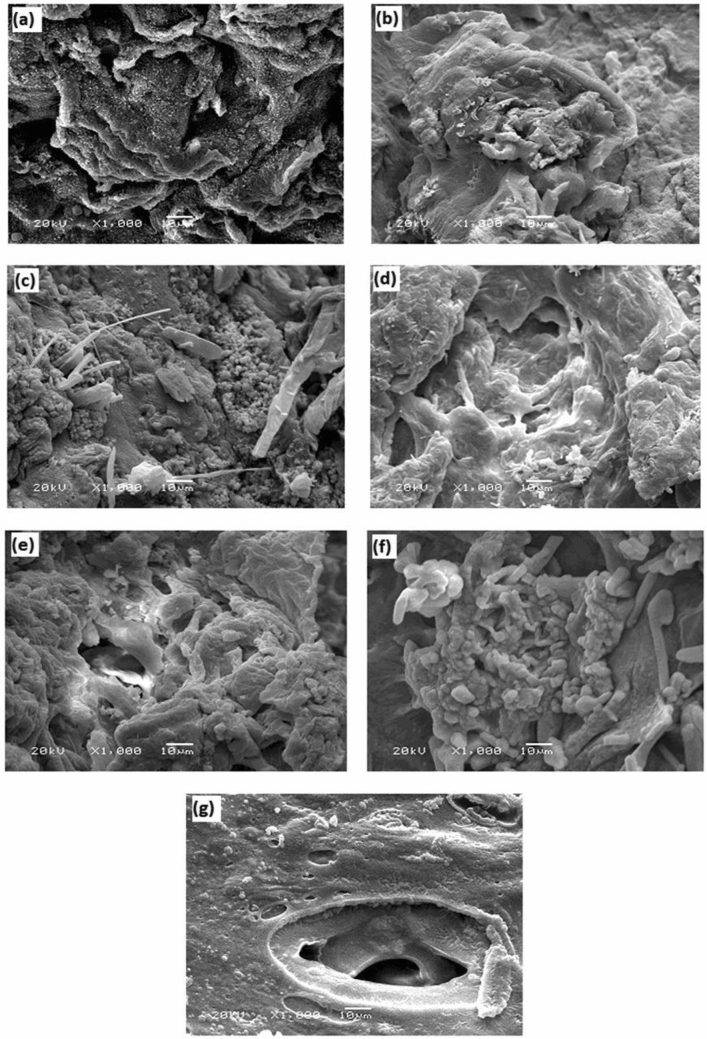
SEM images of (**a**) Ch, (**b**) Ch-Cit (11.25% G), (**c**) Ch-Cit (33.25% G), (**d**) Ch-Cit (46.45% G), (**e**) Ch-Cit (50.0% G), (**f**) Ch-Cit (56.25% G) and (**g**) Ch-Cit (61.0% G) schiff bases.

The surface morphological analysis of chitosan (Ch) and its grafted Schiff base derivatives (Ch-Cit) with different grafting percentages was investigated as shown in Fig. 4a–g. The SEM images show significant changes in the surface morphology with increasing the grafting percentage of citronellal from 11.25 to 61%. The surface morphology of native chitosan displayed rough and irregular surface^37^, while the roughness was increased with increasing the grafting percentage. The SEM image of Ch-Cit (33.25% G) sample shows the appearance of rods and micro particles structures (Fig. 4c). The rods structure started to growth and connected together with the progress of the grafting percentage from 33.25 to 61.0% as demonstrated in Fig. 4d–g. These changes could be a result of the acquired variant of amphiphilic character that generated from the hydrophilic-hydrophobic balance of the chitosan and the newly formed Ch-Cit schiff base. Moreover, the water content plays an important role in the arrangement of the polymers chains, and affects the formed polymer structures accordingly. The heterogeneity of the reaction of aldehyde and the amino groups of chitosan backbone in addition to the polarity difference between chitosan and citronellal could be also an explanation of the roughness character increment^38^.

#### BET analysis

**Figure 5:**
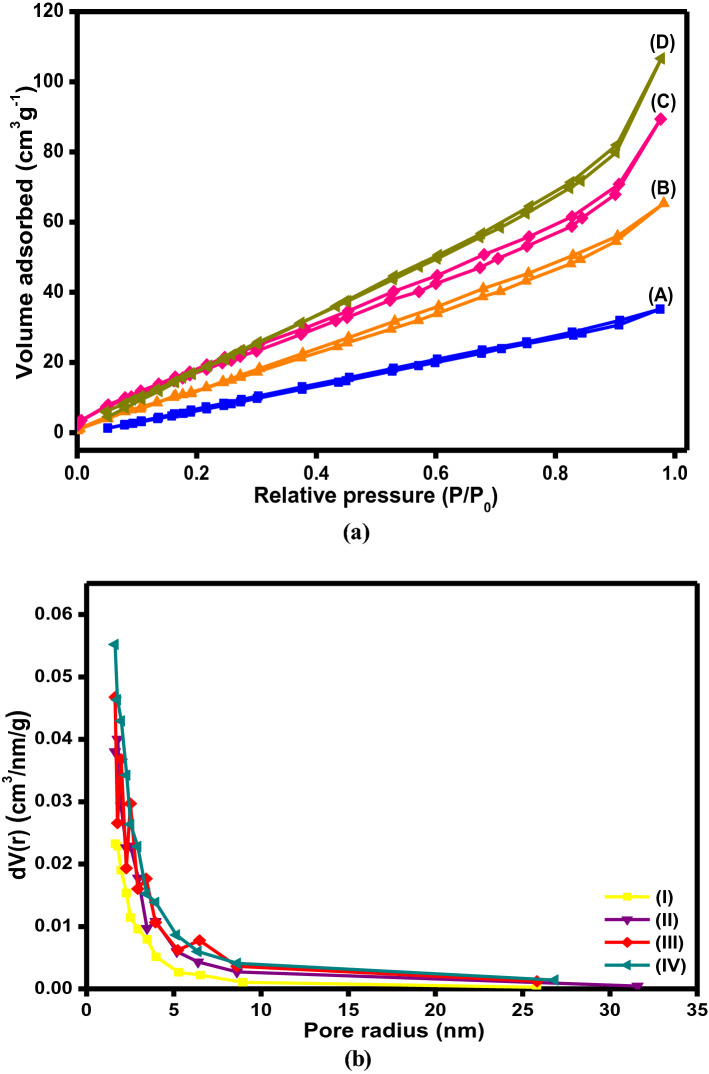
BET analysis of (**a**) N2 adsorption/desorption isotherm of (A) Ch, (B) Ch-Cit (11.25% G), (C) Ch-Cit (46.45% G) and (D) Ch-Cit (61.0% G) schiff bases. (**b**) Pore size distributions of (I) Ch, (II) Ch-Cit (11.25% G), (III) Ch-Cit (46.45% G) and (IV) Ch-Cit (61.0% G) schiff bases.

BET measurements were conducted to scrutinize the impact of different proportions of citronellal on the specific surface area (S_BET_) of chitosan. Three Ch-Cit schiff base samples with different grafting (%) values were analyzed and the results were compared to native chitosan. The N_2_ adsorption/desorption isotherms (Fig. 5a) clarify that chitosan and chitosan-*g*-citronellal schiff base exhibit type II. Besides, the S_BET_ of native Ch, Ch-Cit (11.25% G), Ch-Cit (46.45% G) and Ch-Cit (61.0% G) schiff bases were 57.78, 68.89, 79.68 and 115.94 m^2^/g, respectively. These results demonstrated an apparent boosting in the S_BET_ of the chitosan schiff bases compared to the pristine chitosan. Moreover, The BJH pore size distributions (Fig. 5b) evinced the mesopores structures of native Ch, Ch-Cit schiff bases, since the pore radius of Ch-Cit (11.25% G), Ch-Cit (46.45% G) and Ch-Cit (61.0% G) schiff bases were 3.23, 3.50 and 3.71 nm, while native Ch sample recorded 2.20 nm.

### Optimization of chitosan-g-citronellal schiff base formation

#### Effect of citronellal concentration

**Figure 6:**
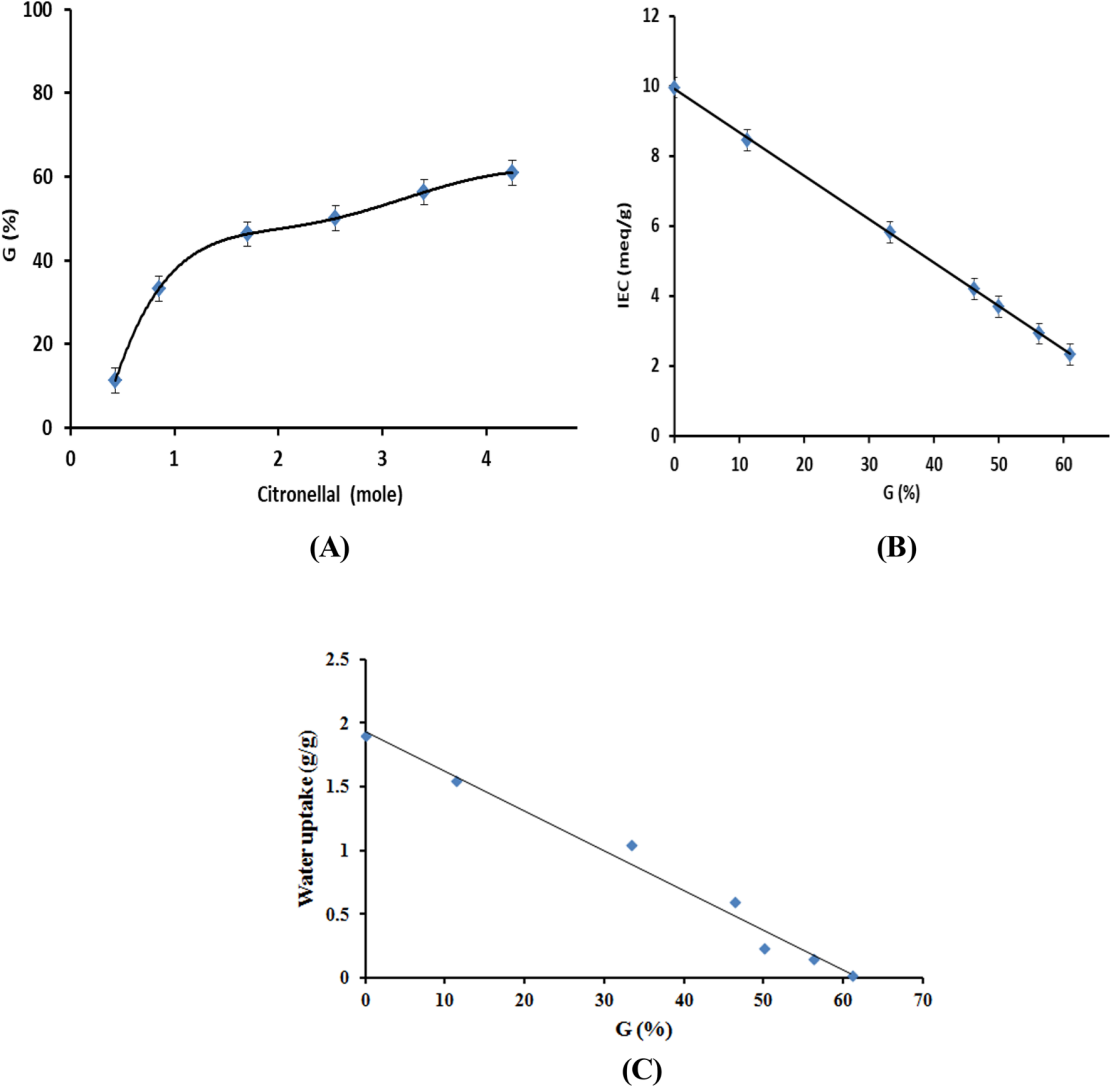
(**A**) Effect of the citronellal concentration on the grafting percentage (G%) of Ch-Cit schiff base, (**B**) Effect of the G% on the IEC values and (**C**) Effect of G% on water uptake profiles.

Figure 6A shows the effect of variation the citronellal (Cit) concentration on the formation of chitosan-*g*-citronellal schiff base (Ch-Cit) through monitoring of the grafting percentage (G %). It is clear that the G (%) value increase within three stages. The first stage was observed with increasing the citronellal concentration from 0.43 to 0.85 mol, since the G (%) value was increased from 11.25 to 33.25%. The second stage was observed with further increase of the reacted citronellal amount up to 1.7 mol, since the G (%) value was increased with a lower rate till reaches the maximum value of 46.45%. The third stage started with increase of the reacted citronellal amount from 2.55 to 4.25 mol, where the G (%) value was increased with the lowest rate to reaches a maximal of 61%. To better understanding the grafting behavior, we have to point out the following hypotheses;

1. The reaction between the citronellal and chitosan can be expressed by a click-reaction technique.
2. The variation of the grafting percentage refers to the difference in the ability of the citronellal molecules towards the schiff base formation with chitosan.
3. The grafting of citronellal on to chitosan backbone is monolayer.
4. The grafting process started in homogenous conditions, where all the reactants are soluble. On contrast, the progress formation of the chitosan-*g*-citronellal schiff base, which is a hydrophobic and less soluble in the reaction medium, the heterogeneous conditions took place.
5. The water uptake capacity of the developed chitosan-*g*-citronellal schiff base became lesser with the progress of the grafting process, and accordingly, reduced the ability of free citronellal molecules to reach the free amine groups of chitosan.

Such explanations were agreed with the obtained solubility results, ion exchange capacity and water uptake results of the developed chitosan-*g*-citronellal schiff base.

Table 1 shows two general observations, the first is that the solubility of the Ch-Cit schiff base samples was decreased with increasing the grafting percentage over the studied pH range. Second, the results clarified that the Ch-Cit sample with the highest G (%) value of 61% turned to be almost insoluble at the studied pH range; 3.0–8.0. On the other hand, the ion exchange capacity (IEC) profiles show inversely proportional linear relation (Fig. 6B). The IEC value of chitosan (Ch) recorded the maximum of 9.95 meq/g, while it decreased gradually with increasing the G (%) value till reached the lowest value of 2.3 meq/g for Ch-Cit (61.0% G) schiff base derivative. These results could be explained by increasing the affinity formation of the chitosan-*g*-citronellal schiff base between the citronellal aldehyde groups and amino groups of chitosan, which in turn leads to the consumption of amine groups and leads consequently to decrease of its ion exchange capacity. Similar profiles have been attained by the author’s previous studies^39^.

**Table 1:** Solubility (%) values of chitosan and chitosan-g-citronellal schiff base at different pHs.

pH	Ch (0.0% G)	Ch-Cit (11.25% G)	Ch-Cit (33.25% G)	Ch-Cit (46.25% G)	Ch-Cit (50.0% G)	Ch-Cit (56.25% G)	Ch-Cit (61.0% G)
3.0	99.98 ± 2.1	99.88 ± 1.4	99.66 ± 1.2	88.77 ± 2.6	65.17 ± 1.4	49.58 ± 1.4	1.99 ± 1.4
4.0	99.95 ± 1.6	99.62 ± 1.9	87.21 ± 3.2	72.21 ± 2.7	43.67 ± 1.4	21.76 ± 1.4	1.75 ± 1.4
5.0	96.42 ± 3.1	83.59 ± 2.4	53.67 ± 1.4	27.23 ± 2.4	17.92 ± 1.4	9.80 ± 1.4	0.78 ± 1.4
6.0	90.78 ± 3.3	81.74 ± 2.6	51.27 ± 1.3	39.59 ± 1.7	12.76 ± 1.4	7.52 ± 1.4	0.57 ± 0.9
7.0	0	0	0	0	0	0	0
8.0	0	0	0	0	0	0	0

Besides, the water uptake (WU) profiles of Ch and Ch-Cit schiff base samples were investigated as presented in Fig. 6C. The obtained results display a remarkable decrease of water uptake values of Ch-Cit schiff base samples compared to native chitosan. In addition, the values were decreased linearly from 1.55 to 0.02 g/g with increasing the grafting percentage from 11.25 to 61%, while the highest value of 1.9 g/g was attained by native chitosan sample. These results could be attributed to changing the hydrophilic nature of chitosan to hydrophobic one via the grafting and schiff base formation^40^.

### Evaluation of oil spill removal process

#### Impact of oil type

**Figure 7:**
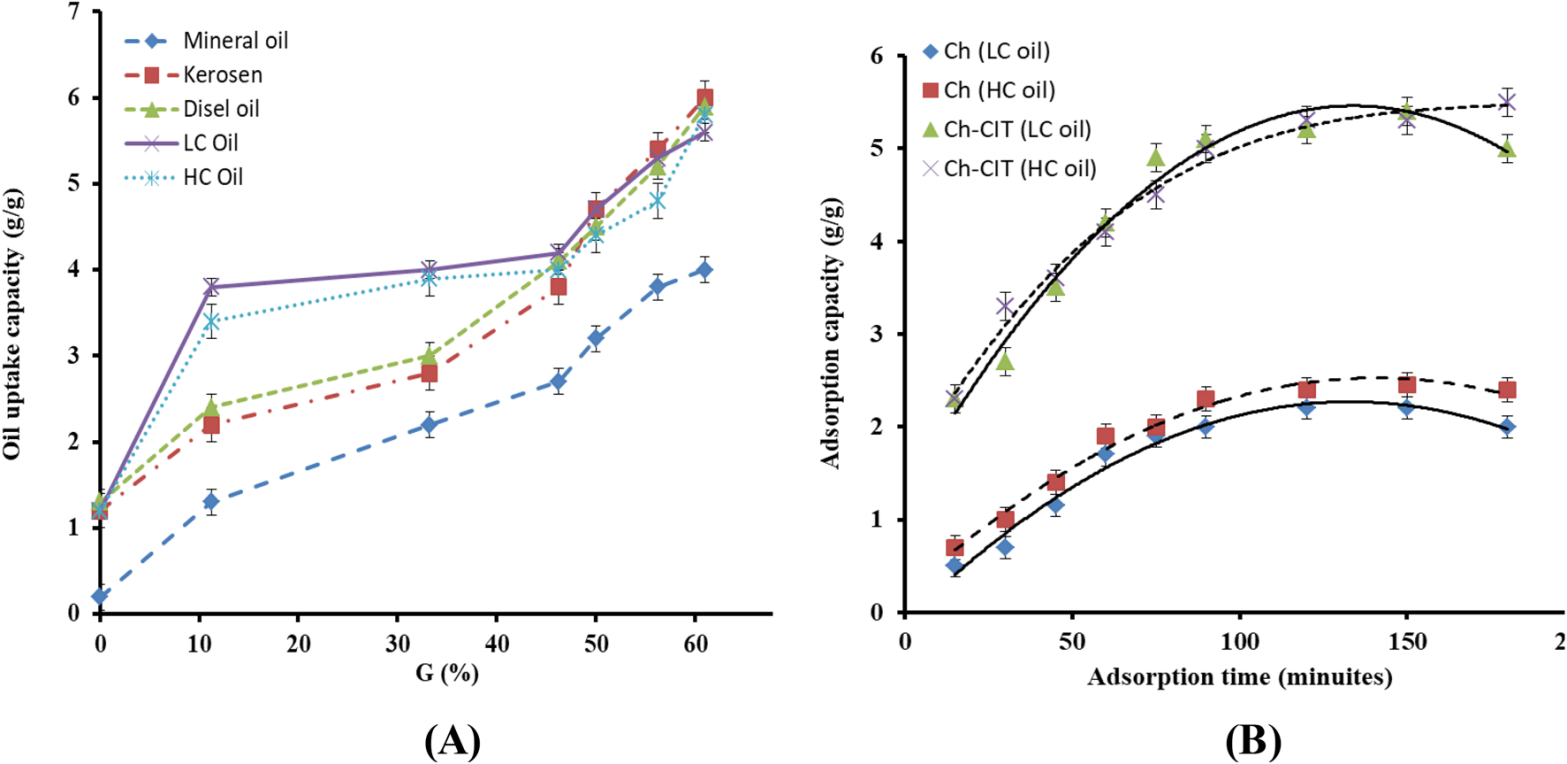
(**A**) Effect of the grafting percentage (G%) on the uptake capacity of different oils and (**B**) Effect of adsorption time on the oil adsorption capacity of Ch and Ch-Cit schiff base using LC and HC oils.

The developed chitosan-*g*-citronellal schiff base amphiphilic polymer has been tested its oil uptake capacity towards four different oils types, namely; mineral oil, kerosene, diesel oil, light crude (LC) oil and heavy crude (HC)oil as depicted in Fig. 7A. The results clarified that the prepared Ch-Cit schiff base demonstrated higher oil uptake capacity values for all studied oils types compared with native chitosan. In addition, it can be seen that the higher values were attained by the highly viscous oils. Basically, high viscosity can improve the adherent forces between oil molecules and the hydrophobic adsorbent surface^13,40^. In addition, an exponentially increase of the oil capacity values was observed with increasing the grafting percentages, since adsorbent with the highest grafting percentage (61%) has the highest oil uptake capacity value (6 g/g). Increasing the hydrophobic character caused by the presence of hydrocarbon long chains of citronellal in addition to the occurred reduction in the free hydrophilic amine groups of chitosan significantly enhances the oil sorption uptake and then increasing its oil uptake values consequently. Accordingly, light and heavy crude oils were selected for performing further adsorption investigations.

#### Impact of adsorption time

Figure 7B reveals that the adsorption time has a linear positive impact on the oil adsorption capacity for both Ch and Ch-Cit schiff base until 90 min. Beyond 90 min, the adsorption capacity increment rate started to be lower and leveled off after 120 min. The adsorption capacity of the Ch-Cit schiff base samples towards both LC and HC oils was greatly improved compared to native Ch. The adsorption capacity leveling off after 120 min of adsorption time referred to two opposite reasons^41^. The first concerns the adsorbent, where all of the adsorption sites over the adsorbent surface were consumed and the interior pores were filled due to the repellence forces amongst the oils at bulk and phases. Thus, the affinity of adsorbent active sites towards oil molecules reduced, and subsequently, the oil-desorption process befallen. The gained results agreed with the other reported studies^23,42^. The second reason concerns the available oil amounts. For the native Ch adsorbent, the first reason is the determined factor, since only 40% of the oil amount was removed after 120 min. On the other side, the second reason is the determined factor for the Ch-Cit schiff base adsorbents, where 98% of the oil amounts were removed after 120 min. This behavior is expected due to the hydrophobic nature of the Ch-Cit schiff base adsorbents which enable them to attach oil molecules easily on their surface as well as inside their porous structures. Therefore, the number of attached molecules increased with increasing the contact time, and this in turn led to a great enhancement in the adsorption capacity^43^.

#### Impact of oil amount

**Figure 8:**
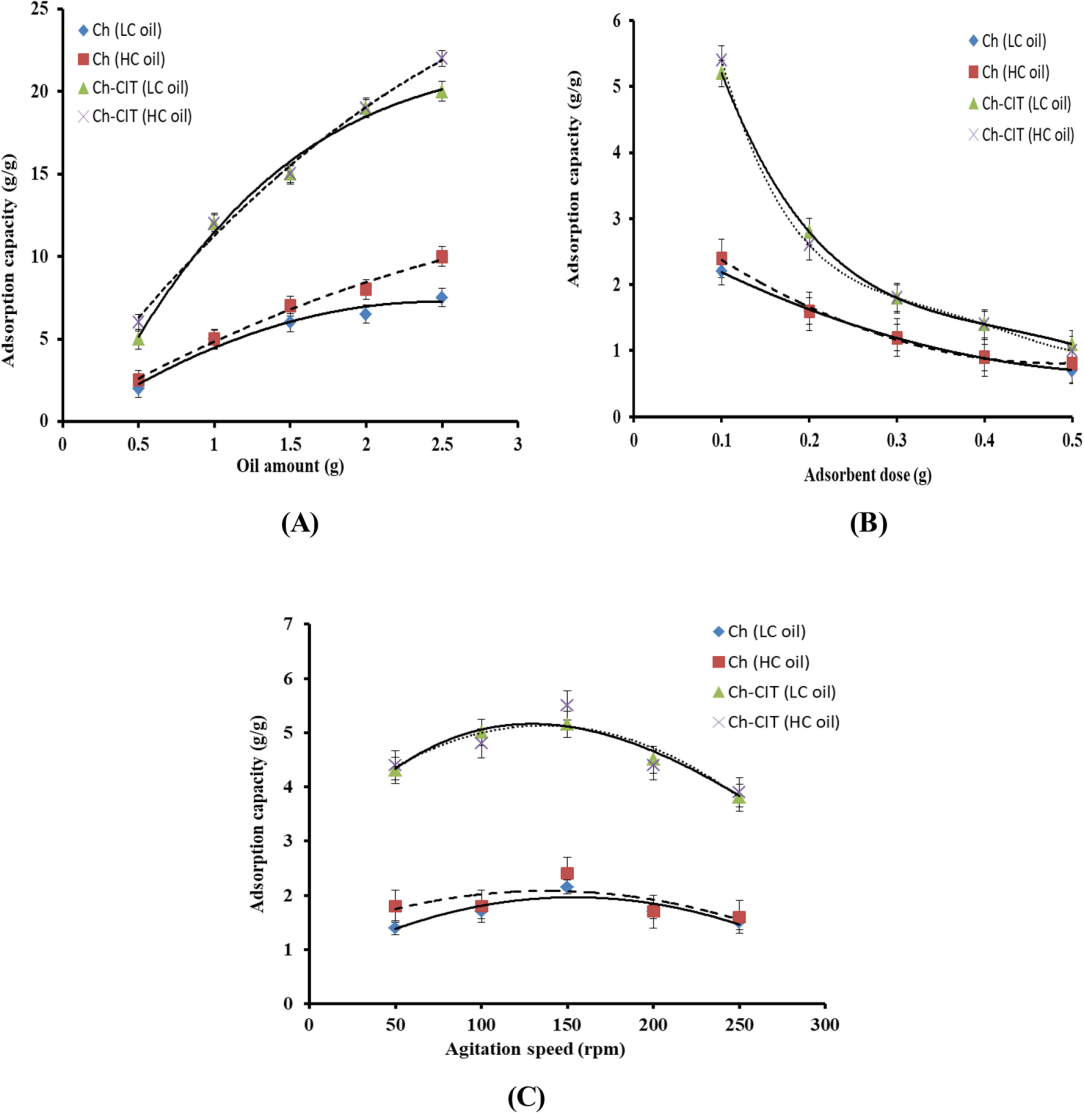
(**A**) Effect of oil amount, (**B**) effect of adsorbent dose and (**C**) effect of agitation speed on the oil adsorption capacity of Ch and Ch-Cit schiff base using LC and HC oils.

To explore the maximum potentials of the developed adsorbent matrix, the effect of oil amount variation from 0.5 to 2.5 g was studied as presented in Fig. 8A. A direct relation between the oil amount and the adsorption capacity has been noticed for Ch and Ch-Cit schiff base adsorbents. The adsorption capacity towards the LC oil becomes lower than the HC oil using oil amount beyond 2.0 g. At the same time, the adsorption capacity of the Ch-Cit schiff base adsorbent is higher than three folds of the adsorption capacity of native Ch. Generally, a concentration gradient between the oil–water liquid phase and the adsorbent solid phase creates a driving force necessary to overcome all resistances of the oil between the aqueous and solid phases. Moreover, higher number of oil molecules is available to the adsorbent sites which enhance the sorption capacity^44^.

#### Impact of adsorbent dose

Variation the adsorbent dose from 0.1 to 0.5 g has a significant impact on the adsorption profiles as shown in Fig. 8B. The adsorption capacity of native Ch linearly decreased with increasing the adsorbent dose, while the decrease was exponentially in case of the Ch-Cit schiff base matrix. This decline is fundamentally attributed to the higher number of the available adsorption sites on the developed Ch-Cit schiff base adsorbent relative to the native chitosan counterpart. Consequently, increasing the adsorbent dose at a constant crude oil amount leads directly to a reduction in the adsorbed oil amount per unit mass of the adsorbent as a result of the residence of a limited amount of oil per unit mass of the adsorbent^13,40^. It is worthy to mention that only 0.1 g of the Ch-Cit schiff base adsorbent can remove 96 and 98.98% of the light and heavy crude oils, respectively.

#### Impact of agitation speed

The effect of variation the agitation speed (50–250 rpm) on the adsorption capacity was studied as depicted in Fig. 8C. The results clarify that that increasing the agitation speed from 50 to 150 rpm has a positive outcome on the adsorption profile of Ch and Ch-Cit schiff base adsorbents. These observations could be attributed to the increase in the turbulence as the agitation speed increases up to 150 rpm, which in turn reduces the thickness of the boundary oil-layer around the surface of the adsorbent. Accordingly, the diffusion rate of the spilt oil towards the surface of the adsorbent increases and the exposed surface area of the adsorbent also increases, which leads to an increase in the adsorption capacity. Nevertheless, a decline in the oil adsorption profile was observed with a further increase in the agitation speed beyond 150 rpm, since the adsorption capacity was reduced by about 30% at 250 rpm. Indeed, the higher agitation speed produces an oil**–**water emulsion process, which weakens the attraction forces between the oil molecules and the adsorption sites on the surface of the adsorbent. This in turn leads to deformation of the stationary film and the oil-desorption process took place accordingly^40^.

#### Optimization using response surface methodology (RSM)

The adsorption processes are presented by Box–Behnken design, which identify the superlative levels of the variables as time (min), amount of oil (g) and temperature (°C) as illustrated in Tables 2 and 3 for heavy crude oil (HC oil) adsorption using native Ch and Ch-Cit schiff base, respectively. The quadratic model demonstrated the statistical relationship between the selected variables and the response in terms of coded factors is the perfect fitted with the following equations:

5 $$ {\text{Y}}_{{{\text{CHa}}}} = {38}.{48} + {9}.{\text{2875X}}_{{1}} - {5}.{\text{92625X}}_{{2}} + {12}.{\text{21125X}}_{{3}} + {1}.{\text{54875X}}_{{1}}^{{2}} + {6}.{8}0{\text{125X}}_{{{21}}} + {4}.{\text{89625X}}_{{3}}^{{2}} - 0.{\text{46X}}_{{1}} {\text{X}}_{{2}} + 0.{\text{1675X}}_{{2}} {\text{X}}_{{3}} + 0.{\text{765X}}_{{1}} {\text{X}}_{{3}}, $$

6 $$ {\text{Y}}_{{{\text{CHa}}/{\text{CIT}}}} = {72}.{36} + {11}.{\text{63X}}_{{1}} - {5}.{3}0{\text{375X}}_{{2}} + {2}.{\text{16375X}}_{{3}} + {2}.{\text{28625X}}_{{1}}^{{2}} + {1}0.0{\text{7375X}}_{{{21}}} + {11}.{\text{42875X}}_{{3}}^{{2}} + {1}.{\text{795X}}_{{1}} {\text{X}}_{{2}} + {1}.{\text{3525X}}_{{2}} {\text{X}}_{{3}} - {1}.{\text{375X}}_{{1}} {\text{X}}_{{3}}, $$

where, Y is the response (Oil adsorption (%)) and X1 is the contact time (min), X2 is the amount of oil (g), and X3 represents the temperature (°C).

**Table 2:** Central composite matrix of real values along with experimental and predicted values for oil adsorption (%) of HC oil using native Ch adsorbent.

Trial	Time (min) (X1)	Amount of oil (g) (X2)	Temp. (°C) (X3)	Oil adsorption (%) of native chitosan (Ch)	
				Measured	Predicted
1	0 (120)	− 1 (0.5)	− 1 (25)	43.25	44.06
2	0 (120)	1 (2.5)	− 1 (25)	32.88	31.87
3	0 (120)	− 1 (0.5)	1 (45)	67.14	68.15
4	0 (120)	1 (2.5)	1 (45)	57.44	56.63
5	− 1 (60)	− 1 (0.5)	0 (35)	42.96	43.01
6	− 1 (60)	1 (2.5)	0 (35)	30.21	32.08
7	1 (180)	− 1 (0.5)	0 (35)	64.37	62.50
8	1 (180)	1 (2.5)	0 (35)	49.78	49.73
9	− 1 (60)	0 (1.5)	− 1 (25)	25.05	24.19
10	− 1 (60)	0 (1.5)	1 (45)	48.14	47.08
11	1 (180)	0 (1.5)	− 1 (25)	40.18	41.24
12	1 (180)	0 (1.5)	1 (45)	66.33	67.19
13	0 (120)	0 (1.5)	0 (35)	38.48	38.48

**Table 3:** Central composite matrix of real values along with experimental and predicted values for oil adsorption (%) of HC oil using Ch-Cit schiff base adsorbent.

Trial	Time (min) (X1)	Amount of oil (g) (X2)	Temperature (°C) (X3)	Oil adsorption (%) of chitosan-*g*-citronellal (Ch-Cit) schiff base	
				Measured	Predicted
1	0 (120)	− 1 (0.5)	− 1 (25)	98.46	98.36
2	0 (120)	1 (2.5)	− 1 (25)	85.56	85.04
3	0 (120)	− 1 (0.5)	1 (45)	99.46	99.98
4	0 (120)	1 (2.5)	1 (45)	91.97	92.07
5	− 1 (60)	− 1 (0.5)	0 (35)	80.64	80.19
6	− 1 (60)	1 (2.5)	0 (35)	66.03	65.99
7	1 (180)	− 1 (0.5)	0 (35)	99.82	99.86
8	1 (180)	1 (2.5)	0 (35)	92.39	92.84
9	− 1 (60)	0 (1.5)	− 1 (25)	70.35	70.91
10	− 1 (60)	0 (1.5)	1 (45)	78.05	77.98
11	1 (180)	0 (1.5)	− 1 (25)	96.85	96.92
12	1 (180)	0 (1.5)	1 (45)	99.05	98.49
13	0 (120)	0 (1.5)	0 (35)	72.36	72.36

**Figure 9:**
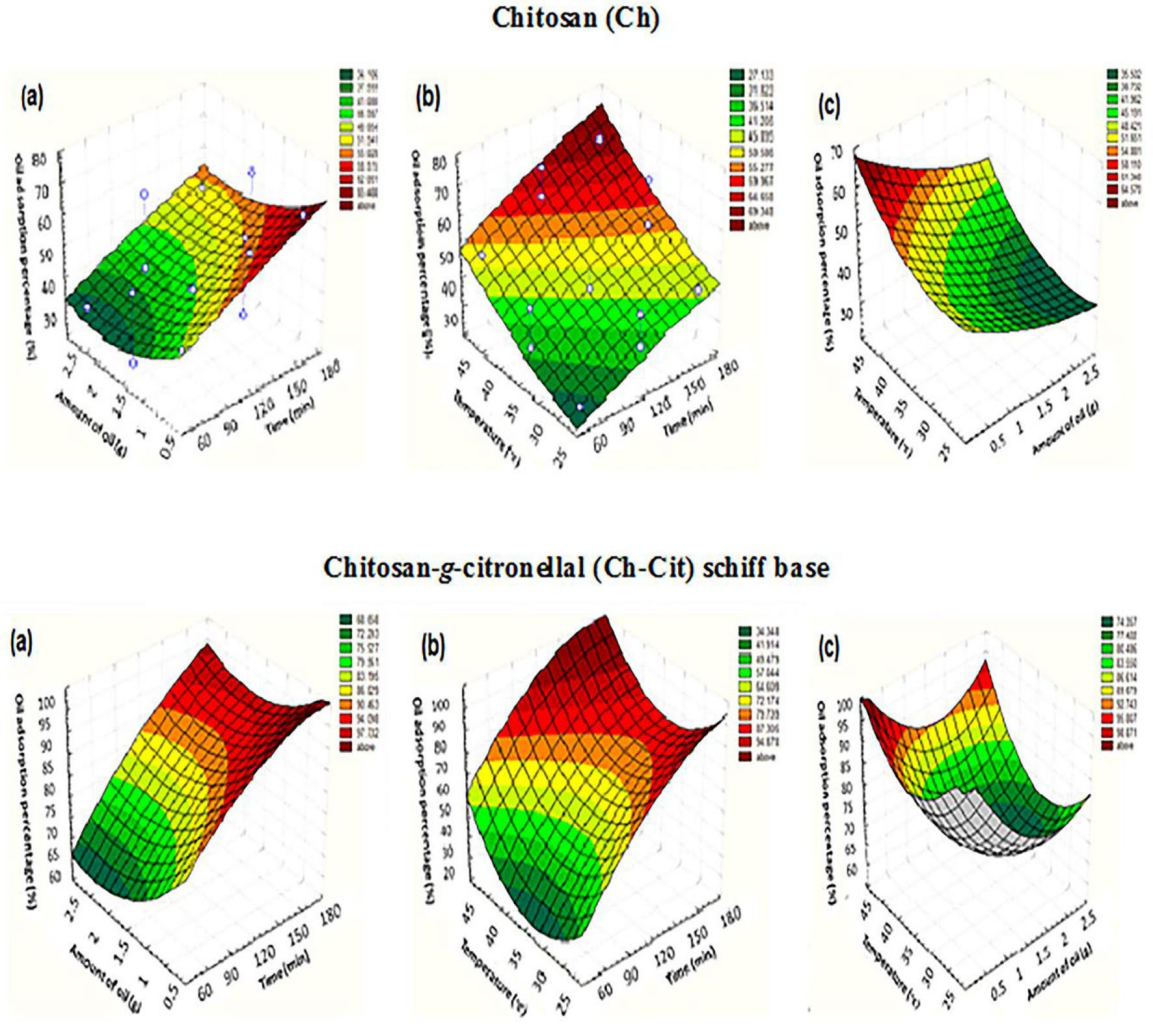
Response surface plots for oil adsorption (%) of heavy crude oil onto chitosan (Ch) and chitosan-*g*-citronellal (Ch-Cit) schiff base adsorbents under effect of (a) time/amount of oil [at dose of adsorbent: 0.1 g; at 25 °C], (b) effect of time/temp [at amount of oil = 0.5 g; at dose of adsorbent: 0.1 g] and (c) effect of temp./amount of oil for [at time:120 min; dose of adsorbent: 0.1 g].

The outcomes of the Box–Behnken design can be offered in 3D presentations with contours. The 3D surface plots demonstrate the type of interaction between the tested variables which allow obtaining the optimum conditions^45^. These plots of the second order polynomial equation with two variables keeping constant and the further two variables within the determined experimental ranges are introduced in Fig. 8 for native Ch and Ch-Cit schiff base. The maximum predicted value is represented by the surface plots. Figure 9 illustrates the simultaneous effect of contact time and amount of oil on heavy crude oil adsorption (%) for native Ch and Ch-Cit schiff base adsorbents. The oil adsorption (%) was found to be decreased with an increase in the amount of oil from 0.5 to 2.5 g, since the maximum value was obtained with 0.5 g of heavy crude oil. After 120 min, the oil adsorption (%) of native Ch lies between 44.06 and 68.15%, while it lies between 98.40 and 99.98%in case of Ch-Cit schiff base adsorbent^46,47^.

On the other hand, the oil adsorption (%) increases with an increase in contact time and then remained roughly constant after 120 min. This behavior revealed that in the beginning, the sorbent materials are adsorbed externally and the adsorption rate increased rapidly, other than, after a certain time the sorbent material become saturated and its affinity towards sorbed molecules decreased but at some point in time, reached a constant value where no more oil is adsorbed from the solution. In addition, the oil adsorption (%) of heavy crude oil for Ch-Cit schiff base adsorbent was higher than native Ch with increasing the contact time. This observation is expected due to the increase of hydrophobic nature of the synthesized schiff base compared to native chitosan, which enable them to attach oil molecules easily onto the adsorbent surface as well as inside their porous structures.

The fitted surface plots oil adsorption (%) versus the combined effect of amount of oil and solution temperature of heavy crude oil for native Ch and Ch-Cit schiff base is also shown. It was appreciated that when the amount of oil is low, the optimum temperature is achieved at lower degree, maybe due to the existence of a smaller number of oil molecules in the solution to be adsorbed on Ch-Cit schiff base. Nevertheless, at the highly amount of oil, the effect of heating is endothermic^48,49^.

In this respect, the solver function of the Microsoft Excel tools was used to estimate the optimal levels of the three components. These optimal levels were obtained from the maximum point of the polynomial model to achieve maximum oil adsorption (%) of 68.15 and 99.98% for Ch and Ch-Cit schiff base adsorbents, respectively at 120 min, 0.5 g amount of oil and solution temperature 45 °C. Therefore, these results give emphasis to the necessity and value of an optimization process.

As shown in Tables 2 and 3 for model validations, the agreement between the predicted and measured oil adsorption (%) demonstrated that using response surface method to design the experiments can be considered as an successful choice in the optimization of the current work parameters besides its uses as an experimental design and statistical analysis.

#### Comparative study

The adsorption capacity of the developed adsorbent was compared with some natural, modified natural and synthetic adsorbents (Table 4)^49–56^. Unmodified chitosan, flakes and powders have very low adsorption capacity, while chitosan from prawn shells has much higher adsorption capacity. Modification of chitosan via grafting with hydrophilic polyacrylamide branches improves the oil adsorption capacity relative to chitosan^22^. Cellulose aerogel based adsorbents functionalized with methyltrimethoxysilane show moderately high adsorption capacity due to the high surface area and the induced hydrophobicity by the grafted methyltrimethoxysilane branches. Grafting cellulose with hydrophobic and oleophilic polymer, butyl acrylate, proved the BuAc cellulose graft copolymer with a moderate adsorption capacity. Acetylated corncobs show very low adsorption capacity regardless the acquired hydrophobicity with acetylation. Carbonized rice husks (CRH) shows slightly low adsorption capacity. On the other hand, the synthetic polymer such as Butyl rubber and Non-woven polypropylene show a moderate adsorption capacity without any modifications. Accordingly, it was evident that the chitosan-*g*-citronellal (Ch-Cit) schiff base apparently exhibit an acceptable performance compared with other natural and modified natural origin adsorbents early reported. However, we did not take into our consideration the diversity of the operating conditions, the effective cost and the reusability.

**Table 4:** Comparison of maximum sorption capacity for the crude oil by various sorbents.

Adsorbent	Sorption capacity	Ref.
Chitosan flakes	0.38 g/g	^49^
Chitosan powder	0.28 g/g	^49^
Chitosan-based polyacrylamide hydrogel	2.31 g/g	^50^
Chitosan (prawn shells)	18.52 g/g	^51^
Acetylated corncobs	0.08 mg/g	^52^
Lauric acid (LA) modified oil palm leaves	1.2 mg/g	^53^
Carbonized rice husks (CRH)	6 g/g	^53^
Cellulose aerogel functionalized with methyltrimethoxysilane	24.41 g/g	^54^
Butyl rubber	25 g/g	^55^
Non-woven polypropylene	15 g/g	^55^
BuAc cellusoe graft copolymer	13.83 g/g	^56^
Chitosan-*g*-citronellal schiff base	24.40 g/g	This study

## Conclusion

The present work was undertaken to prepare new chitosan-*g*-citronellal schiff base adsorbent and evaluate its adsorption aptitude towards crude oil spills. The developed schiff base was characterized under advanced analytical tools such as FT-IR, ^1^H NMR, SEM and BET. Results showed that increasing the amount of citronellal in the feed mixture enhanced the hydrophobic characters for the prepared schiff base derivatives. Furthermore, evaluation of the oil spill adsorption process was also accomplished under different seawater environmental conditions such as: oil type, oil amount, adsorbent dose and contact time. The results revealed that there was a major increase in the oil adsorption capacity of chitosan-*g*-citronellal (Ch-Cit) schiff base compared to the chitosan due to increasing its hydrophobic characters after schiff base formation. Positively, the adsorption capacity of all prepared adsorbents showed acceptable adsorption aptitudes toward crude oils. The optimization using response surface methodology (RSM) proved the agreement between the predicted and measured oil adsorption (%). Therefore, using RSM method for designing the adsorption experiments can be considered as a successful choice in the optimization of the work parameters. The maximum heavy crude oil adsorption (%) were 98.98% for chitosan-*g*-citronellal schiff base compared to 64.99% for native chitosan at constant contact time 180 min, adsorbent amount (0.1 g), oil amount (0.5 g), shaking rate (150 rpm) and adsorption temperature medium (25 ^○^C). The results nominate the developed novel chitosan-*g*-citronellal schiff base as a promising adsorbent for petroleum oil spill removal.
